# LONGITUDINAL ASSOCIATIONS OF SOCIAL CONNECTEDNESS, SOCIAL CONTRIBUTION, SOCIAL ENGAGEMENT, AND LATE-LIFE DRUG USE

**DOI:** 10.1093/geroni/igac059.2018

**Published:** 2022-12-20

**Authors:** Atami De Main, Daniel Powers, Bo Xie

**Affiliations:** Weill Cornell University, New York City, New York, United States; The University of Texas at Austin, Austin, Texas, United States; The University of Texas at Austin, Austin, Texas, United States

## Abstract

Substance misuse among older adults is a growing and complex problem with implications for society, aging adults, and their families. It is understudied in research and clinical practice. Limited and mixed evidence exists that suggests relationships between changes in older adults’ social environment and their drug use over time. This study addressed this gap by examining potential associations between social environment and drug use among community-dwelling older adults. Data were drawn from 3-waves of the national longitudinal survey of Midlife Development in the United States (MIDUS) (N= 2,020; age range=55-94 years; Mean=63.10, SD=5.66 at Wave 1). We estimated multilevel logistic growth models to assess the relationships between social environment indicators (social connectedness, social contribution, social engagement) and drug use, controlling for age, marital status, race/ethnicity, education level, household income, employment status, and number of chronic conditions. Our findings showed significant odds of drug use over the 20-year period of study, which were increased among older adults with multiple chronic conditions with a 59.5% risk of drug-related problems. Social contribution (feelings of being valued by society) negatively predicted older adults’ drug use whereas social connectedness and social engagement were not significant predictors over time. The models also indicated a moderating effect of social contribution on drug use, showing low changes in drug use over time with low social contribution. Our findings illuminate the differential roles of social environment indicators in drug use. Future research, policy, and practice may particularly focus on the role of social contribution in late-life drug use.

